# Profiling of Substrate Specificities of 3C-Like Proteases from Group 1, 2a, 2b, and 3 Coronaviruses

**DOI:** 10.1371/journal.pone.0027228

**Published:** 2011-11-02

**Authors:** Chi-Pang Chuck, Hak-Fun Chow, David Chi-Cheong Wan, Kam-Bo Wong

**Affiliations:** 1 School of Life Sciences, The Chinese University of Hong Kong, Hong Kong, China; 2 Department of Chemistry, The Chinese University of Hong Kong, Hong Kong, China; 3 School of Biomedical Sciences, The Chinese University of Hong Kong, Hong Kong, China; University of Hong Kong, Hong Kong

## Abstract

**Background:**

Coronaviruses (CoVs) can be classified into alphacoronavirus (group 1), betacoronavirus (group 2), and gammacoronavirus (group 3) based on diversity of the protein sequences. Their 3C-like protease (3CL^pro^), which catalyzes the proteolytic processing of the polyproteins for viral replication, is a potential target for anti-coronaviral infection.

**Methodology/Principal Findings:**

Here, we profiled the substrate specificities of 3CL^pro^ from human CoV NL63 (group 1), human CoV OC43 (group 2a), severe acute respiratory syndrome coronavirus (SARS-CoV) (group 2b) and infectious bronchitis virus (IBV) (group 3), by measuring their activity against a substrate library of 19×8 of variants with single substitutions at P5 to P3' positions. The results were correlated with structural properties like side chain volume, hydrophobicity, and secondary structure propensities of substituting residues. All 3CL^pro^ prefer Gln at P1 position, Leu at P2 position, basic residues at P3 position, small hydrophobic residues at P4 position, and small residues at P1' and P2' positions. Despite 3CL^pro^ from different groups of CoVs share many similarities in substrate specificities, differences in substrate specificities were observed at P4 positions, with IBV 3CL^pro^ prefers P4-Pro and SARS-CoV 3CL^pro^ prefers P4-Val. By combining the most favorable residues at P3 to P5 positions, we identified super-active substrate sequences ‘VARLQ↓SGF’ that can be cleaved efficiently by all 3CL^pro^ with relative activity of 1.7 to 3.2, and ‘VPRLQ↓SGF’ that can be cleaved specifically by IBV 3CL^pro^ with relative activity of 4.3.

**Conclusions/Significance:**

The comprehensive substrate specificities of 3CL^pro^ from each of the group 1, 2a, 2b, and 3 CoVs have been profiled in this study, which may provide insights into a rational design of broad-spectrum peptidomimetic inhibitors targeting the proteases.

## Introduction

A number of coronaviruses (CoVs) have been identified as causative agents of respiratory tract and gastroenteritis diseases in mammals and birds [1], [2], [3], [4], [5], [6], [7], [8], [9], [10], [11]. Sequence analysis suggests that these coronaviral strains can be classified into three main groups – alphacoronavirus (group 1), betacoronavirus (group 2), and gamacoronavirus (group 3) [12]. The sequence of severe acute respiratory syndrome coronavirus (SARS-CoV), discovered in 2003, was found to be diverse from any existing groups of CoVs. The group 2 CoVs are then further divided into 2a and 2b sub-groups, with the original group 2 CoVs assigned to group 2a and SARS-CoV to group 2b [13], [14]. Most of coronaviral strains are group 1 and 2a members. They include the four human coronaviruses (HCoVs) strains, NL63, 229E, OC43 and HKU1, that associate with up to 5% of total respiratory tract disease cases [15], [16]. The most infamous strain in group 3 is infectious bronchitis virus (IBV), which can cause lethal infections in birds [17], [18].

3C-like protease (3CL^pro^), which is also named main protease, is responsible for the processing of the viral polyproteins into at least 15 non-structural proteins, most of which are constituents of the viral replication and transcription complex. The cleavage process can be acted in cis and in trans [19]. This enzyme is a good drug target for anti-coronaviral infection, as inhibiting the autocleavage process can inhibit viral replication and reduce virus-induced cytopathic effects on host cells [20], [21], [22], [23]. A detailed knowledge of substrate specificity of 3CL^pro^ is helpful in the rational design of inhibitors. Substrate specificity of SARS-CoV 3CL^pro^ was extensively investigated after the outbreak of SARS in 2003. Fan *et al*. measured the protease activity against 34 single-substituted variants at P5 to P1' positions, while Goetz *et al*. profiled the specificity at P4 to P1 positions by using a fully degenerated library of tetrapeptide mixtures [24], [25]. Chuck *et al*. profiled the substrate preference of SARS-CoV 3CL^pro^ by measuring the activity of 3CL^pro^ against substrate variants with single substitutions at P5 to P3' positions [26].

On the other hand, reports describing the substrate specificities of 3CL^pro^ in group 1, 2a, and 3 are scarce. Only the activity of 3CL^pro^ from HCoV-229E (group 1), transmissible gastroenteritis coronavirus (group 1) and mouse hepatitis virus (group 2a) against three to four of their own autocleavage sequences have been measured by Hegyi *et al*. [27]. Comprehensive study on substrate specificities of group 1, 2a and 3 3CL^pro^ is lacking. Here, we profiled the substrate specificities of selected 3CL^pro^ from group 1, 2a, 2b and 3 CoVs. Activities of 3CL^pro^ from HCoV-NL63 (group 1), HCoV-OC43 (group 2a), SARS-CoV (group 2b) and IBV (group 3) against a substrate library of 19×8 variants were measured by fluorescence resonance energy transfer (FRET) assay [26]. Similarities and differences in substrate specificities among different 3CL^pro^ are discussed.

## Results

### Profiling substrate specificities of 3CL^pro^ from group 1, 2a, 2b, and 3 CoVs

**Figure 1:**
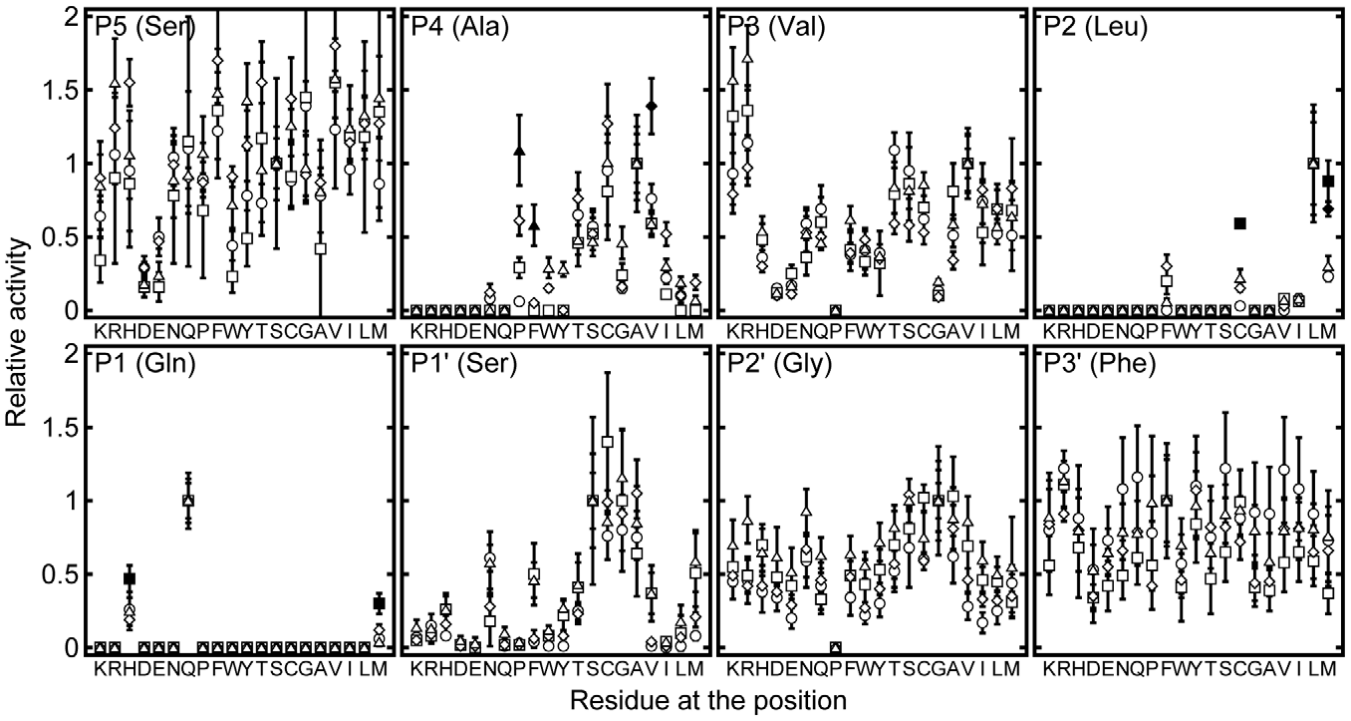
Substrate specificity of 3CL^pro^ at P5 to P3' positions. Relative protease activity of 3CL^pro^ from HCoV-NL63 (circles, group 1), HCoV-OC43 (squares, group 2a), SARS-CoV (diamond, group 2b) and IBV (triples, group 3) against 19×8 of substrate variants were measured by FRET assay. Relative activities that are significantly (p-value<0.001) higher than the rest are represented as filled symbols.

**Figure 2:**
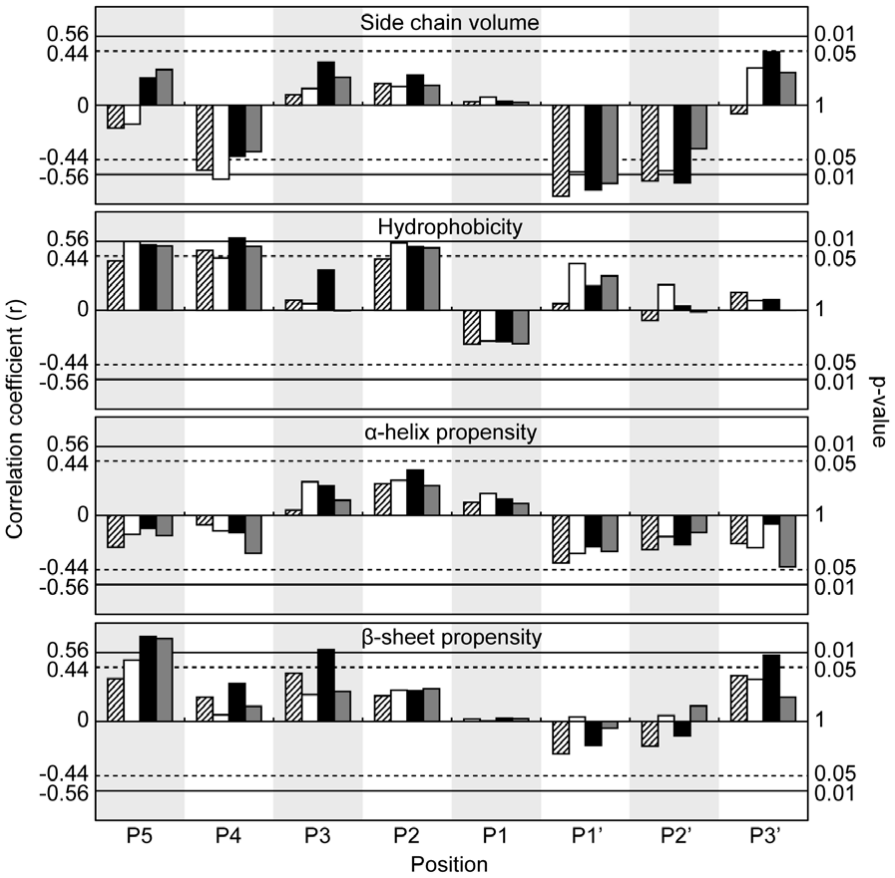
Correlation between 3CL^pro^ activities and structural properties of substituting residues. The relative protease activities of 3CL^pro^ from HCoV-NL63 (shaded, group 1), HCoV-OC43 (white, group 2a), SARS-CoV (black, group 2b) and IBV (grey, group 3), were correlated with structural properties of substituting residue properties, including side chain volume [28], hydrophobicity [29] and α-helix and β-sheet propensities [30]. Correlation coefficients of +/−0.56 and +/−0.44 correspond to p-values of 0.01 and 0.05 respectively.

We have previously created a 19×8 substrate library by performing saturation mutagenesis at P5 to P3' positions on the wild type (WT) sequence (SAVLQ↓SGF), which corresponds to the autocleavage sequence at the N-terminus of SARS-CoV 3CL^pro^ [26]. The values of k_obs_/[3CL^pro^] of the proteases against this WT sequence were 443±11, 124±13, 180±5 and 174±19 mM^-1^ min^-1^ for HCoV-NL63 (group 1), HCoV-OC43 (group 2a), SARS-CoV (group 2b), and IBV (group 3), respectively. That all proteases can cleave the WT sequence efficiently justifies that we can use our substrate library to profile the substrate specificities of 3CL^pro^ from other groups of CoVs. Based on the FRET assay we developed, we measured the activities of 3CL^pro^ from HCoV-NL63, HCoV-OC43, SARS-CoV and IBV against the 19×8 substrate variants (Figure 1, Table S1) [26]. To identify the structural basis of substrate preferences for different CoVs, the protease activities were correlated with side chain volume [28], hydrophobicity [29], and α-helix and β-sheet propensities [30] as described [26]. The correlations were quantified in terms of correlation coefficients and p-values (Figure 2, Table S2).

### Differences in substrate specificities among 3CL^pro^

We then tested if the relative activities of 3CL^pro^ from any CoV strains were significantly different from the other by analysis of variance. Substitutions that resulted in significantly higher relative activities (p<0.001) were indicated as filled symbol in Figure 1. IBV 3CL^pro^ (Figure 1, triangles) was the most efficient in cleaving A4P and A4F with relative activities of 1.09±0.24 and 0.58±0.14, respectively, while SARS 3CL^pro^ (Figure 1, diamonds) preferred A4V with relative activity of 1.39±0.19. HCoV-OC43 3CL^pro^ (Figure 1, squares) appeared to be the most versatile in accepting substitutions at P1 and P2 positions, and could cleave Q1H, Q1M, L2M and L2C, significantly better than 3CL^pro^ from other strains. No significant differences were observed for other substitutions, suggesting that 3CL^pro^ from different CoVs shares many similarities in substrate preferences.

### Substrate preferences that are common to all 3CL^pro^

The most preferred P1 residue is Gln (Figure 1), which forms hydrogen-bonds with the side-chain of an invariant His residue and the backbone carbonyl group of an invariant Phe residue (His-163 and Phe-140 in SARS-CoV 3CL^pro^) in the P1 binding pocket. Interestingly, our results showed that 3CL^pro^ from all groups of CoVs can cleave His at P1 position reasonably well. The relative activities for 3CL^pro^ from HCoV-NL63, HCoV-OC43, SARS-CoV, and IBV were 0.26±0.08, 0.47±0.08, 0.19±0.03 and 0.25±0.12, respectively (Table S1). Consistent with this observation, His is found natively at P1 positions in the polyproteins from group 1 and 2a CoVs (Table S3). Taken together, the ability to cleave His at P1 position is a conserved property for all 3CL^pro^. Moreover, we showed that all 3CL^pro^ can cleave Q1M, albeit at an even lower rate, and all other substitutions resulted in undetected activity.

The protease activities correlate positively with the hydrophobicity of substituting residues at P2 position (Figure 2). In fact, among the P2 variants, only L2M, L2C, L2F, L2I and L2V were cleavable, suggesting that P2 position favors hydrophobic residues. However, substitution with β-branched residues, Val or Ile, led to >10-folds decreases in the activity (Figure 1, Table S1). Considering that Leu, Val and Ile share similar hydrophobicity and side chain volume, the large differences in activities suggest that β-branched residues are not preferred in all 3CL^pro^, probably due to steric clashes with the P2 binding pocket. Taken together, P2 position prefers hydrophobic residues without β-branch, and the most preferred residue is Leu.

At P3 position, the protease activities on Arg/Lys-substituting variants were 5 to 14 fold higher than that on Asp/Glu-substituting variants (Figure 1, Table S1). This observation suggests that P3 position prefers positively charged residues over negatively charged one. In the active site of 3CL^pro^, there is no substrate-binding pocket for P3 residue. Molecular modeling showed that there is an invariant Glu residue (Glu-166 in SARS-CoV 3CL^pro^) in the active site of 3CL^pro^ that may form favorable charge-charge interactions with a positively charged residue at the P3 position, which may explain why Arg/Lys are favored over Asp/Glu at this position (Figure S1). Moreover, no cleavage was observed for substrate containing Pro-substitution at P3 position.

**Figure 3:**
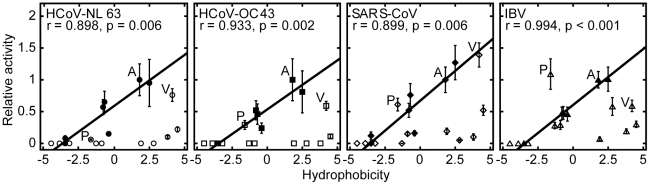
All 3CL^pro^ prefer small hydrophobic residues at P4 position. All 3CL^pro^ activities are highly correlated to hydrophobicity of residues with side chain volumes of <70 Å^3^ (filled symbols). The correlation coefficients and the corresponding p-values are indicated.

The protease activities correlate negatively with side chain volume, and positively with the hydrophobicity of substituting residues at P4 position (Figure 2). The correlations with hydrophobicity were more evident (with correlation coefficients >0.89) when only small residues (Ala, Asn, Asp, Cys, Gly, Ser, and Thr) with side chain volumes <70 Å^3^ (Figure 3) were included in the analysis. This result suggests that as long as the side chain can fit into the P4 binding pocket, the protease activity is directly proportional to the hydrophobicity of the substituting residues. On the other hand, charged residues like Lys, Arg, His, Asp and Glu were not cleavable, presumably due to the unfavorable burial of charges in the hydrophobic P4 pocket.

In general, the activities of 3CL^pro^ correlate positively with the hydrophobicity and β-sheet propensity of substituting residues at P5 position (Figure 2). The correlations are significant (p<0.05) for group 2a, 2b, and 3 CoVs, but are weaker for group 1 CoV. Like the P3 position, there is no substrate-binding pocket for P5 residue. In the crystal structure of SARS-CoV 3CL^pro^ in complex with a peptide substrate, the P5 residue adopts an extended β-strand conformation to avoid clashing of P5-P6 residues with the protease [31]. Residues with high β-sheet propensity may stabilize the extended conformation at P5 and improve enzyme-substrate interaction. As shown in Figure 1, a number of substitutions at P5 position resulted in a substrate better than the WT sequence (i.e. with relative activity >1). Consistent with the suggestion that P5 position favors residues with high hydrophobicity and β-sheet propensity, Val-substitution consistently yielded substrates with higher than WT activities for all 3CL^pro^. On the other hand, negatively charged residues (Asp/Glu) were not favored at P5 position, with significantly lower activities (0.16 to 0.50).

At P1' position, the protease activities correlate negatively with side chain volume of substituting residues (Figure 2). In fact, the relative activities for substrates with the smallest residues (Gly, Ala, Ser, and Cys) at P1' position were in the range of 0.64 to 1.40, which were consistently higher than those for other larger residues (Figure 1). At P2' position, all variants, except G2'P, could be cleaved with relative activities of 0.17 to 1.04 (Figure 1). The protease activities also correlate negatively with the side chain volume (Figure 2), but the difference in the protease activities was relatively small (Figure 1). At P3' position, no obvious substrate preference was observed.

### The effect of combining multiple favorable substitutions

Our profiling analysis showed that all CoV 3CL^pro^ prefer P5-Val and P3-Arg (Figure 1). To test if we can combine two favorable substitutions to create a more active substrate, we have created a doubly-substituted substrate variant ‘VARLQ↓SGF’. The protease activities of HCoV-NL63, HCoV-OC43, SARS-CoV and IBV against the doubly-substituted sequence were 1.70±0.07, 1.87±0.17, 1.70±0.12 and 3.24±0.37, respectively (Table 1). The results suggest that the increase in activity is additive, and the sequence ‘VARLQ↓SGF’ can represent a good broad-spectrum substrate for all 3CL^pro^.

**Table 1:** 3CL^pro^ activities against doubly- and triply-substituted substrate variants. WT substrate was substituted at P3 to P5 positions to generate doubly- and triply-substituted variants. The relative activities of 3CL^pro^ on these substrate variants are reported.

Variant sequence	HCoV-NL63	HCoV-OC43	SARS-CoV	IBV
VAVLQ↓SGF	1.23±0.40	1.55±0.30	1.80±0.31	1.58±0.27
SARLQ↓SGF	1.14±0.24	1.36±0.17	0.97±0.12	1.72±0.22
VARLQ↓SGF	1.70±0.07	1.87±0.17	1.70±0.17	3.24±0.37
SPVLQ↓SGF	0.06±0.01	0.29±0.07	0.61±0.10	1.09±0.24
VPRLQ↓SGF	0.15±0.04	0.91±0.12	0.99±0.12	4.33±0.98
SVVLQ↓SGF	0.76±0.10	0.59±0.07	1.39±0.19	0.59±0.09
VVVLQ↓SGF	1.23±0.06	0.60±0.05	1.97±0.19	0.86±0.05
VVRLQ↓SGF	1.63±0.07	0.55±0.04	2.50±0.51	2.19±0.13

On the other hand, our profiling analysis suggests that 3CL^pro^ from SARS-CoV and IBV have different substrate preferences at P4 position – SARS-CoV prefers P4-Val (relative activity = 1.09±0.24) while IBV prefers P4-Pro (relative activity = 1.39±0.10) (Figure 1, Table S1). To see if we can exploit this distinct substrate preference at P4 position to create a substrate more specific for IBV 3CL^pro^, we have created the triply-substituted variant ‘VPRLQ↓SGF’. The protease activity of IBV 3CL^pro^ against this sequence was boosted to 4.33±0.98, while that of the other strains were significantly reduced, demonstrating that this substrate sequence can represent a specific substrate-sequence for IBV 3CL^pro^ (Table 1). Similarly, the protease activity of SARS-CoV 3CL^pro^ against the triply-substituted sequence ‘VVRLQ↓SGF’ was boosted to 2.50±0.51, while that of the other strains were reduced (Table 1). Taken together, these results suggest that one can combine the substrate preference profiled in this study to create a better substrate sequences.

## Discussion

This study provides the first comprehensive profiling of substrate specificities of 3CL^pro^ from group 1, 2a, and 3 CoVs. We showed that the substrate specificities of these 3CL^pro^ share many similarities to those of 3CL^pro^ from SARS-CoV (group 2b) reported previously by us [26]. Table 2 summarizes the substrate specificities that are common to all 3CL^pro^. Although the substrate specificities for 3CL^pro^ from different groups of CoVs share a number of similarities, unique substrate preferences were identified in this study. In particular, we showed that only IBV 3CL^pro^, but not other proteases, prefers P4-Pro (Figure 3).

**Table 2:** Summary of substrate specificities that are common among all 3CL^pro^.

Position	Substrate preferences
P5	No strong preference
P4	Small hydrophobic residues
P3	Positively charged residues
P2	High hydrophobicity and absence of β-branch
P1	Gln
P1'	Small residues
P2'	Small residues
P3'	No strong preference

**Figure 4:**
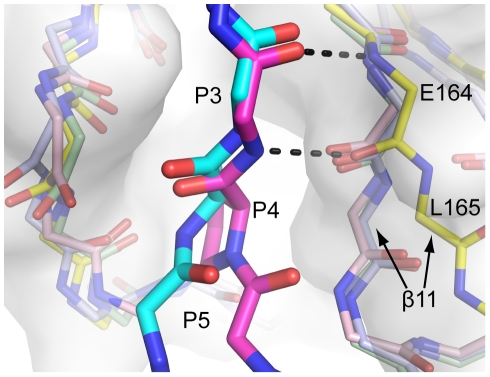
IBV 3CL^pro^ has a wider substrate-binding pocket to accommodate substrate containing P4-Pro. The structure of IBV 3CL^pro^ (PDB: 2Q6D, yellow cartoon and white surface) is superimposed with 3CL^pro^ from HCoV-229E (PDB: 1P9S, light blue), HCoV-HKU1 (PDB: 3D23, light green), and SARS-CoV (PDB: 2Q6G, pink) [31], [32], [33]. The structure of WT substrate (magenta) is derived from crystal structure of SARS-CoV 3CL^pro^ in complex with the autocleavage sequence (TSAVLQ↓SGFRKM) (PDB: 2Q6G) [31]. The structure of the A4P substrate variant (cyan) was modeled based on the crystal structure of IBV 3CL^pro^ in complex with its own autocleavage sequence (PDB: 2Q6D) [31]. Note that strand-11 of IBV 3CL^pro^ is positioned further away from P4 to P5 positions, resulting in a wider substrate-binding pocket.

To understand the structural basis of this unique substrate preference, we compared the structures of IBV 3CL^pro^ with other coronaviral 3CL^pro^. We noticed that strand-11 of IBV 3CL^pro^ is positioned further away from the P4 and P5 substrate-binding site compared to other 3CL^pro^ (Figure 4) [31], [32], [33]. This results in a wider substrate-binding pocket in IBV 3CL^pro^. We further docked the substrate variant A4P into the substrate-binding pocket of IBV 3CL^pro^. Due to the cyclic structure of Pro residue, the backbone Ø dihedral angle of the P4 residue is restrained to ca. −60°, which causes the substrate peptide to bend towards the strand-11 of 3CL^pro^. Such conformation of substrate is much better accommodated by IBV 3CL^pro^, which has a wider substrate-binding pocket near the P4 and P5 positions. This observation justifies why only IBV 3CL^pro^ cleaves P4-Pro efficiently.

Similarities in substrate specificity suggest that it is feasible to create a broad-spectrum inhibitor that targets all 3CL^pro^. A broad-spectrum inhibitor is desirable for a first line defense against coronaviral infection because CoVs are capable of generating novel strains with high virulence through high frequency of mutations and recombination [34], [35], [36], [37].. Based on the autocleavage sequence of SARS-CoV 3CL^pro^ (i.e. AVLQ↓), Rao and co-workers designed broad-spectrum peptidomimetic inhibitors that can inhibit 3CL^pro^ from different groups of CoVs [20]. Their results are consistent with our observation that the autocleavage sequence of SARS-CoV 3CL^pro^ can be well cleaved by all 3CL^pro^. The substrate preferences profiled in this study will provide a rational basis to improve the broad-spectrum 3CL^pro^ inhibitors. For example, by combining favorable substitutions at P3 to P5 positions, we identified a substrate sequence ‘VARLQ↓SGF’ that can be cleaved with high relative activities by 3CL^pro^ from all groups of CoVs (Table 1). This substrate sequence may serve as a good starting point of the design of broad-spectrum peptidomimetic inhibitors for 3CL^pro^.

Although it is generally accepted that substrate specificity provides insights into the design of peptidomimetic protease inhibitors, there are exceptions to the dogma that good peptidomimetic inhibitors should be derived from good substrate sequences. For example, Hilgenfeld and co-workers showed that the P2 position of peptide aldehyde inhibitors can accommodate aspartate or serine, which are poor substrates for SARS-CoV 3CL^pro^ [38].

In the FRET assay developed by us, all 3CL^pro^ can efficiently cleave the WT sequence of ‘SAVLQ↓SGF’ with activity of 120–440 mM^−1^ min^−1^, and the activity can be further improved by 1.7 to 3.2 fold using the substrate sequence of ‘VARLQ↓SGF’. Because the substrate sequences can be cleaved by all 3CL^pro^ with high efficiency, one could use the FRET assay to screen for broad-spectrum inhibitors targeting 3CL^pro^ from all groups of CoVs.

## Materials and Methods

### Cloning, Expression and Purification of 3CL^pro^ and the Substrate Library

Cloning, expression and purification of SARS-CoV 3CL^pro^ were described previously [26]. Codon-optimized DNA sequences encoding HCoV-NL63 (GenBank AY567487) and HCoV-OC43 (GenBank AAX85666), and IBV (GenBank M95169) 3CL^pro^ were purchased from Mr. Gene (http://mrgene.com). The coding sequences of 3CL^pro^ from HCoV-NL63, HCoV-OC43 and IBV were sub-cloned and expressed in *E. coli* strain BL21 (DE3) pLysS as fusion proteins with N-terminal tags of poly-histidine-small ubiquitin-related modifier (His_6_-SUMO) or poly-histidine-maltose binding protein (His_6_-MBP). Protein expression was induced by addition of 0.1 mM of isopropyl β-D-1-thiogalactopyranoside. After overnight incubation at 25°C, cells were harvested by centrifugation and resuspended in buffer A (20 mM Tris, pH 7.8, 150 mM NaCl and 1 mM tris(2-carboxyethyl)phosphine) with 30 mM imidazole and disrupted by sonication. Soluble fraction was subject to immobilized metal ion affinity chromatography for purification as described for SARS-CoV 3CL^pro^ [26]. The His_6_-SUMO or His_6_-MBP tags were removed by protease digestion using sentrin-specific protease 1 or factor Xa, respectively, followed by immobilized metal ion affinity chromatography. Native 3CL^pro^ were finally purified by G75 size exclusion column and stored in buffer A. Elution profiles of size exclusion chromatography indicated that all 3CL^pro^ purified were dimeric.

The construction, expression and purification of the substrate library were described previously [26]. In brief, the WT substrate sequence ‘TSAVLQ↓SGFRKM’ was inserted between the cyan fluorescent protein and the yellow fluorescent protein to create the substrate protein. Saturation mutagenesis was performed at each of the P5 to P3' positions to generate a substrate library of 19×8 variants.

### FRET assay for 3CL^pro^ activity measurement

The protease activity of 3CL^pro^ was measured by the FRET assay we developed previously [26]. Purified 3CL^pro^ at 0.2 to 2 µM were mixed with 35 µM of the substrate protein in buffer A. Cleavage of the substrate protein leads to a decrease in fluorescence at 530 nm when the reaction mixture was excited at 430 nm. The fluorescence intensity, monitored by EnVision 2101 Multilabel Plate Reader, was fitted to single exponential decay to obtain the observed rate constant (k_obs_). The protease activity against variant substrates was normalized against the WT activity to yield the relative activity. The assay was repeated in triplicate.

### Correlation analysis

Structural properties of substituting residues, including side chain volume [28], hydrophobicity [29], and α-helix and β-sheet propensities [30], were correlated with relative activity to determine correlation coefficients (r) and p-values.

## Supporting Information

Figure S1 **Molecular modeling showing P3-Arg may interact with Glu-166 of 3CL^pro^.** The model was based on the crystal structure of 3CLpro (grey) in complex with a peptide substrate ‘TSAVLQ↓SGFRK’ (yellow). P3-Val was replaced by P3-Arg using the program PyMOL. As shown, the invariant Glu-166 is in close proximity to P3-Arg, and may form favorable charge-charge interaction to P3-Arg.
(TIF)

Table S1 Relative activities of HCoV-NL63, HCoV-OC43, SARS-CoV and IBV 3CL^pro^. ND stands for non-detectable cleavage. The average and the standard deviation of three measurements are shown.
(DOC)

Table S2 Correlation between activity of 3CL^pro^ and structural properties of substituting residues. The correlation coefficients and p-values (bracketed) are reported.
(DOC)

Table S3 Autocleavage sequences of 3CL^pro^. PEDV, TGEV, MHV, PHEV stand for porcine epidemic diarrhoea coronavirus, transmissible gastroenteritis coronavirus, mouse hepatitis coronavirus and porcine hemagglutinating encephalomyelitis coronavirus respectively.
(DOC)
