# Supplementary figures and images for: Profiling of Substrate Specificities of 3C-Like Proteases from Group 1, 2a, 2b, and 3 Coronaviruses
Source: PLoS One. 2011 Nov 2;6(11):e27228. doi: 10.1371/journal.pone.0027228 (PMC3206940; doi:10.1371/journal.pone.0027228)

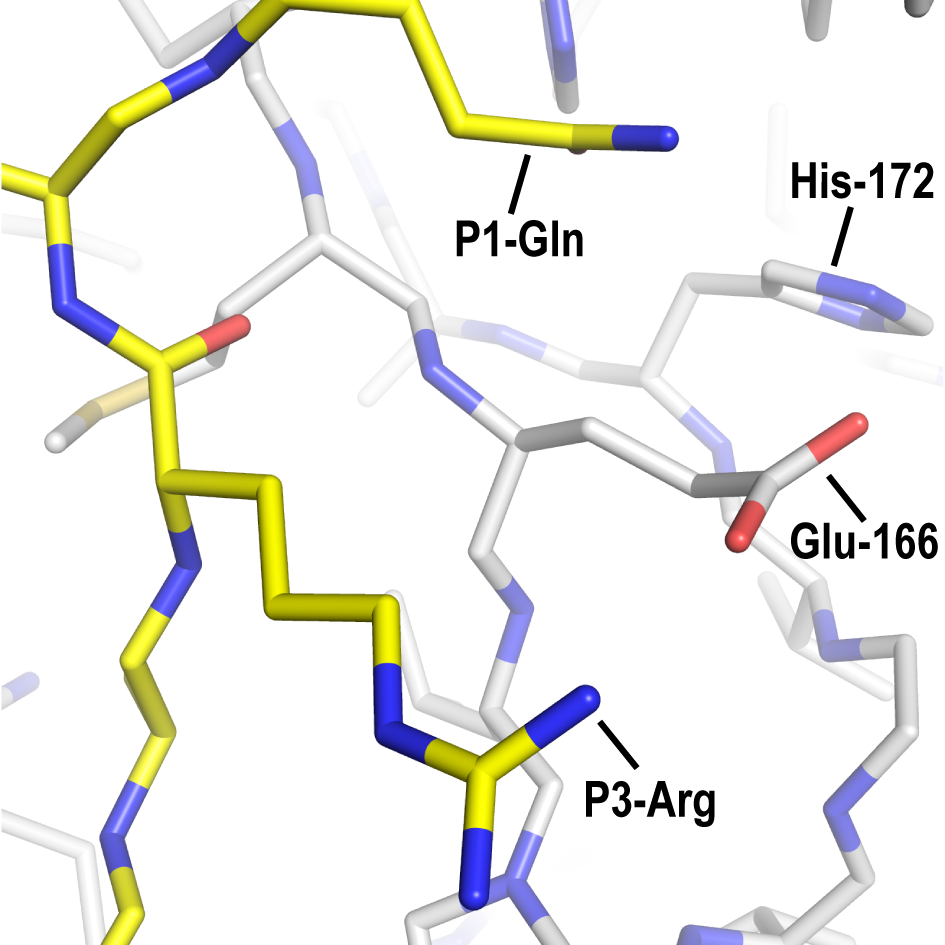

Supplement: Figure S1 — Molecular modeling showing P3-Arg may interact with Glu-166 of 3CLpro. The model was based on the crystal structure of 3CLpro (grey) in complex with a peptide substrate ‘TSAVLQ↓SGFRK’ (yellow). P3-Val was replaced by P3-Arg using the program PyMOL. As shown, the invariant Glu-166 is in close proximity to P3-Arg, and may form favorable charge-charge interaction to P3-Arg. (TIF) [file pone.0027228.s001.tif]
