# Supplementary material for: Profiling of Substrate Specificities of 3C-Like Proteases from Group 1, 2a, 2b, and 3 Coronaviruses
Source: PLoS One. 2011 Nov 2;6(11):e27228. doi: 10.1371/journal.pone.0027228 (PMC3206940; doi:10.1371/journal.pone.0027228)
Supplement: Table S1 — Relative activities of HCoV-NL63, HCoV-OC43, SARS-CoV and IBV 3CLpro. ND stands for non-detectable cleavage. The average and the standard deviation of three measurements are shown. (DOC) [file pone.0027228.s002.doc]

**Table S1.** Relative activities of HCoV-NL63, HCoV-OC43, SARS-CoV and IBV 3CL^pro^. ND stands for non-detectable cleavage. The average and the standard deviation of three measurements are shown.

| **Substrate variant** | **HCoV-NL63** | **HCoV-OC43** | **SARS-CoV** | **IBV** |
| --- | --- | --- | --- | --- |
| S5A | 0.78 ± 0.38 | 0.42 ± 0.55 | 0.87 ± 0.05 | 0.81 ± 0.28 |
| S5C | 0.88 ± 0.17 | 0.91 ± 0.22 | 1.44 ± 0.28 | 1.26 ± 0.11 |
| S5D | 0.29 ± 0.08 | 0.16 ± 0.07 | 0.29 ± 0.04 | 0.18 ± 0.03 |
| S5E | 0.50 ± 0.13 | 0.16 ± 0.10 | 0.47 ± 0.05 | 0.24 ± 0.09 |
| S5F | 1.22 ± 0.17 | 1.36 ± 0.56 | 1.70 ± 0.08 | 1.48 ± 0.56 |
| S5G | 1.39 ± 0.17 | 1.45 ± 0.72 | 0.92 ± 0.14 | 0.97 ± 0.22 |
| S5H | 0.95 ± 0.41 | 0.86 ± 0.43 | 1.55 ± 0.16 | 1.06 ± 0.30 |
| S5I | 0.96 ± 0.16 | 1.19 ± 0.51 | 1.14 ± 0.11 | 1.24 ± 0.25 |
| S5K | 0.64 ± 0.14 | 0.34 ± 0.15 | 0.90 ± 0.16 | 0.85 ± 0.30 |
| S5L | 1.28 ± 0.18 | 1.18 ± 0.65 | 1.27 ± 0.18 | 1.33 ± 0.30 |
| S5M | 0.86 ± 0.16 | 1.35 ± 0.74 | 1.27 ± 0.09 | 1.45 ± 0.28 |
| S5N | 1.04 ± 0.15 | 0.78 ± 0.46 | 0.99 ± 0.14 | 0.89 ± 0.26 |
| S5P | 0.89 ± 0.12 | 0.68 ± 0.46 | 0.87 ± 0.06 | 1.07 ± 0.25 |
| S5Q | 1.10 ± 0.39 | 1.15 ± 0.85 | 0.90 ± 0.07 | 0.92 ± 0.26 |
| S5R | 1.06 ± 0.20 | 0.90 ± 0.58 | 1.24 ± 0.21 | 1.55 ± 0.30 |
| WT | 1.00 ± 0.17 | 1.00 ± 0.58 | 1.00 ± 0.04 | 1.00 ± 0.25 |
| S5T | 0.73 ± 0.13 | 1.17 ± 0.66 | 1.55 ± 0.14 | 0.96 ± 0.22 |
| S5V | 1.23 ± 0.40 | 1.55 ± 0.30 | 1.80 ± 0.31 | 1.58 ± 0.27 |
| S5W | 0.44 ± 0.10 | 0.23 ± 0.11 | 0.91 ± 0.07 | 0.72 ± 0.16 |
| S5Y | 0.78 ± 0.31 | 0.49 ± 0.19 | 1.12 ± 0.24 | 1.43 ± 0.25 |
| WT | 1.00 ± 0.25 | 1.00 ± 0.33 | 1.00 ± 0.20 | 1.00 ± 0.13 |
| A4C | 0.95 ± 0.37 | 0.81 ± 0.33 | 1.27 ± 0.27 | 1.01 ± 0.19 |
| A4D | ND | ND | ND | ND |
| A4E | ND | ND | ND | ND |
| A4F | ND | ND | 0.05 ± 0.01 | 0.58 ± 0.14 |
| A4G | 0.15 ± 0.03 | 0.24 ± 0.08 | 0.16 ± 0.04 | 0.46 ± 0.11 |
| A4H | ND | ND | ND | ND |
| A4I | 0.22 ± 0.04 | 0.11 ± 0.03 | 0.52 ± 0.08 | 0.30 ± 0.05 |
| A4K | ND | ND | ND | ND |
| A4L | 0.10 ± 0.02 | ND | 0.10 ± 0.05 | 0.19 ± 0.04 |
| A4M | ND | ND | 0.19 ± 0.05 | 0.07 ± 0.03 |
| A4N | 0.08 ± 0.04 | ND | 0.12 ± 0.06 | ND |
| A4P | 0.06 ± 0.01 | 0.29 ± 0.07 | 0.61 ± 0.10 | 1.09 ± 0.24 |
| A4Q | ND | ND | ND | ND |
| A4R | ND | ND | ND | ND |
| A4S | 0.57 ± 0.12 | 0.52 ± 0.15 | 0.52 ± 0.11 | 0.47 ± 0.10 |
| A4T | 0.65 ± 0.17 | 0.46 ± 0.16 | 0.76 ± 0.18 | 0.48 ± 0.10 |
| A4V | 0.76 ± 0.10 | 0.59 ± 0.07 | 1.39 ± 0.19 | 0.59 ± 0.09 |
| A4W | ND | ND | 0.15 ± 0.02 | 0.29 ± 0.07 |
| A4Y | ND | ND | ND | 0.28 ± 0.05 |

| **Substrate variant** | **HCoV-NL63** | **HCoV-OC43** | **SARS-CoV** | **IBV** |
| --- | --- | --- | --- | --- |
| V3A | 0.51 ± 0.08 | 0.81 ± 0.19 | 0.34 ± 0.06 | 0.59 ± 0.06 |
| V3C | 0.62 ± 0.09 | 0.70 ± 0.16 | 0.53 ± 0.08 | 0.86 ± 0.08 |
| V3D | 0.15 ± 0.02 | 0.12 ± 0.03 | 0.10 ± 0.02 | 0.12 ± 0.01 |
| V3E | 0.17 ± 0.02 | 0.25 ± 0.06 | 0.11 ± 0.02 | 0.17 ± 0.02 |
| V3F | 0.38 ± 0.05 | 0.41 ± 0.14 | 0.39 ± 0.07 | 0.62 ± 0.09 |
| V3G | 0.13 ± 0.02 | 0.10 ± 0.02 | 0.09 ± 0.01 | 0.20 ± 0.01 |
| V3H | 0.36 ± 0.05 | 0.48 ± 0.11 | 0.30 ± 0.04 | 0.54 ± 0.10 |
| V3I | 0.74 ± 0.15 | 0.53 ± 0.22 | 0.82 ± 0.05 | 0.73 ± 0.27 |
| V3K | 0.93 ± 0.27 | 1.32 ± 0.25 | 0.79 ± 0.07 | 1.57 ± 0.22 |
| V3L | 0.52 ± 0.07 | 0.69 ± 0.13 | 0.69 ± 0.17 | 0.57 ± 0.05 |
| V3M | 0.51 ± 0.24 | 0.68 ± 0.20 | 0.83 ± 0.34 | 0.64 ± 0.23 |
| V3N | 0.59 ± 0.11 | 0.36 ± 0.12 | 0.53 ± 0.15 | 0.52 ± 0.12 |
| V3P | ND | ND | ND | ND |
| V3Q | 0.69 ± 0.16 | 0.60 ± 0.17 | 0.50 ± 0.08 | 0.46 ± 0.05 |
| V3R | 1.14 ± 0.24 | 1.36 ± 0.17 | 0.97 ± 0.12 | 1.72 ± 0.22 |
| V3S | 0.95 ± 0.26 | 0.86 ± 0.25 | 0.58 ± 0.11 | 0.82 ± 0.06 |
| V3T | 1.09 ± 0.12 | 0.79 ± 0.22 | 0.59 ± 0.07 | 0.84 ± 0.25 |
| WT | 1.00 ± 0.20 | 1.00 ± 0.24 | 1.00 ± 0.19 | 1.00 ± 0.10 |
| V3W | 0.41 ± 0.13 | 0.33 ± 0.09 | 0.48 ± 0.08 | 0.42 ± 0.14 |
| V3Y | 0.39 ± 0.06 | 0.32 ± 0.22 | 0.35 ± 0.05 | 0.37 ± 0.03 |
| L2A | ND | ND | ND | ND |
| L2C | 0.03 ± 0.01 | 0.58 ± 0.13 | 0.15 ± 0.02 | 0.22 ± 0.06 |
| L2D | ND | ND | ND | ND |
| L2E | ND | ND | ND | ND |
| L2F | ND | 0.20 ± 0.09 | 0.30 ± 0.08 | 0.06 ± 0.02 |
| L2G | ND | ND | ND | ND |
| L2H | ND | ND | ND | ND |
| L2I | 0.08 ± 0.02 | 0.06 ± 0.02 | 0.06 ± 0.02 | 0.08 ± 0.03 |
| L2K | ND | ND | ND | ND |
| WT | 1.00 ± 0.28 | 1.00 ± 0.40 | 1.00 ± 0.28 | 1.00 ± 0.35 |
| L2M | 0.23 ± 0.03 | 0.88 ± 0.14 | 0.69 ± 0.05 | 0.30 ± 0.07 |
| L2N | ND | ND | ND | ND |
| L2P | ND | ND | ND | ND |
| L2Q | ND | ND | ND | ND |
| L2R | ND | ND | ND | ND |
| L2S | ND | ND | ND | ND |
| L2T | ND | ND | ND | ND |
| L2V | ND | 0.08 ± 0.03 | 0.03 ± 0.01 | 0.07 ± 0.02 |
| L2W | ND | ND | ND | ND |
| L2Y | ND | ND | ND | ND |

| **Substrate variant** | **HCoV-NL63** | **HCoV-OC43** | **SARS-CoV** | **IBV** |
| --- | --- | --- | --- | --- |
| Q1A | ND | ND | ND | ND |
| Q1C | ND | ND | ND | ND |
| Q1D | ND | ND | ND | ND |
| Q1E | ND | ND | ND | ND |
| Q1F | ND | ND | ND | ND |
| Q1G | ND | ND | ND | ND |
| Q1H | 0.26 ± 0.08 | 0.47 ± 0.08 | 0.19 ± 0.03 | 0.25 ± 0.12 |
| Q1I | ND | ND | ND | ND |
| Q1K | ND | ND | ND | ND |
| Q1L | ND | ND | ND | ND |
| Q1M | 0.06 ± 0.09 | 0.30 ± 0.07 | 0.12 ± 0.04 | 0.04 ± 0.03 |
| Q1N | ND | ND | ND | ND |
| Q1P | ND | ND | ND | ND |
| WT | 1.00 ± 0.06 | 1.00 ± 0.13 | 1.00 ± 0.08 | 1.00 ± 0.05 |
| Q1R | ND | ND | ND | ND |
| Q1S | ND | ND | ND | ND |
| Q1T | ND | ND | ND | ND |
| Q1V | ND | ND | ND | ND |
| Q1W | ND | ND | ND | ND |
| Q1Y | ND | ND | ND | ND |
| S1’A | 0.75 ± 0.13 | 0.64 ± 0.29 | 1.05 ± 0.23 | 0.85 ± 0.25 |
| S1’C | 0.76 ± 0.16 | 1.40 ± 0.47 | 0.99 ± 0.08 | 0.86 ± 0.11 |
| S1’D | 0.03 ± 0.01 | 0.02 ± 0.02 | 0.01 ± 0.01 | 0.05 ± 0.03 |
| S1’E | ND | ND | 0.01 ± 0.01 | 0.03 ± 0.04 |
| S1’F | 0.04 ± 0.01 | 0.50 ± 0.21 | 0.06 ± 0.06 | 0.46 ± 0.12 |
| S1’G | 0.80 ± 0.14 | 1.00 ± 0.48 | 0.91 ± 0.13 | 1.16 ± 0.33 |
| S1’H | 0.08 ± 0.02 | 0.26 ± 0.11 | 0.16 ± 0.06 | 0.26 ± 0.09 |
| S1’I | 0.00 ± 0.01 | 0.04 ± 0.02 | 0.01 ± 0.01 | 0.04 ± 0.02 |
| S1’K | 0.07 ± 0.02 | 0.05 ± 0.03 | 0.05 ± 0.01 | 0.14 ± 0.05 |
| S1’L | 0.01 ± 0.01 | 0.10 ± 0.12 | 0.07 ± 0.06 | 0.18 ± 0.11 |
| S1’M | 0.08 ± 0.03 | 0.51 ± 0.27 | 0.21 ± 0.07 | 0.59 ± 0.21 |
| S1’N | 0.61 ± 0.09 | 0.18 ± 0.17 | 0.28 ± 0.12 | 0.58 ± 0.21 |
| S1’P | 0.03 ± 0.01 | 0.02 ± 0.01 | 0.02 ± 0.01 | 0.04 ± 0.01 |
| S1’Q | 0.01 ± 0.01 | 0.02 ± 0.01 | 0.02 ± 0.01 | 0.10 ± 0.04 |
| S1’R | 0.15 ± 0.02 | 0.08 ± 0.04 | 0.09 ± 0.05 | 0.13 ± 0.10 |
| WT | 1.00 ± 0.19 | 1.00 ± 0.57 | 1.00 ± 0.19 | 1.00 ± 0.32 |
| S1’T | 0.25 ± 0.06 | 0.41 ± 0.23 | 0.23 ± 0.07 | 0.43 ± 0.15 |
| S1’V | 0.01 ± 0.02 | 0.37 ± 0.19 | 0.04 ± 0.01 | 0.37 ± 0.14 |
| S1’W | 0.01 ± 0.01 | 0.08 ± 0.03 | 0.08 ± 0.02 | 0.12 ± 0.03 |
| S1’Y | 0.01 ± 0.01 | 0.22 ± 0.10 | 0.08 ± 0.02 | 0.27 ± 0.06 |

| **Substrate variant** | **HCoV-NL63** | **HCoV-OC43** | **SARS-CoV** | **IBV** |
| --- | --- | --- | --- | --- |
| G2’A | 0.62 ± 0.18 | 1.03 ± 0.27 | 0.81 ± 0.16 | 0.88 ± 0.21 |
| G2’C | 0.60 ± 0.03 | 1.02 ± 0.09 | 0.59 ± 0.06 | 0.75 ± 0.17 |
| G2’D | 0.34 ± 0.09 | 0.48 ± 0.14 | 0.38 ± 0.04 | 0.62 ± 0.20 |
| G2’E | 0.20 ± 0.07 | 0.42 ± 0.09 | 0.29 ± 0.07 | 0.52 ± 0.16 |
| G2’F | 0.34 ± 0.12 | 0.49 ± 0.13 | 0.48 ± 0.11 | 0.64 ± 0.12 |
| WT | 1.00 ± 0.37 | 1.00 ± 0.27 | 1.00 ± 0.12 | 1.00 ± 0.21 |
| G2’H | 0.38 ± 0.14 | 0.70 ± 0.14 | 0.42 ± 0.07 | 0.65 ± 0.16 |
| G2’I | 0.17 ± 0.07 | 0.46 ± 0.09 | 0.33 ± 0.05 | 0.60 ± 0.12 |
| G2’K | 0.45 ± 0.12 | 0.55 ± 0.13 | 0.49 ± 0.05 | 0.70 ± 0.17 |
| G2’L | 0.25 ± 0.09 | 0.45 ± 0.10 | 0.32 ± 0.07 | 0.50 ± 0.12 |
| G2’M | 0.44 ± 0.12 | 0.31 ± 0.07 | 0.35 ± 0.15 | 0.55 ± 0.34 |
| G2’N | 0.59 ± 0.18 | 0.62 ± 0.14 | 0.67 ± 0.09 | 0.93 ± 0.15 |
| G2’P | ND | ND | ND | ND |
| G2’Q | 0.42 ± 0.16 | 0.33 ± 0.10 | 0.46 ± 0.08 | 0.63 ± 0.12 |
| G2’R | 0.46 ± 0.16 | 0.49 ± 0.11 | 0.42 ± 0.05 | 0.87 ± 0.16 |
| G2’S | 0.68 ± 0.27 | 0.81 ± 0.16 | 1.04 ± 0.11 | 1.00 ± 0.15 |
| G2’T | 0.52 ± 0.14 | 0.70 ± 0.23 | 0.57 ± 0.16 | 0.82 ± 0.15 |
| G2’V | 0.28 ± 0.09 | 0.69 ± 0.15 | 0.46 ± 0.08 | 0.86 ± 0.17 |
| G2’W | 0.22 ± 0.06 | 0.43 ± 0.17 | 0.27 ± 0.04 | 0.56 ± 0.09 |
| G2’Y | 0.30 ± 0.09 | 0.53 ± 0.14 | 0.40 ± 0.04 | 0.72 ± 0.13 |
| F3’A | 0.91 ± 0.32 | 0.39 ± 0.14 | 0.45 ± 0.10 | 0.59 ± 0.19 |
| F3’C | 0.88 ± 0.13 | 0.99 ± 0.22 | 0.72 ± 0.12 | 0.94 ± 0.09 |
| F3’D | 0.53 ± 0.28 | 0.34 ± 0.17 | 0.35 ± 0.08 | 0.53 ± 0.17 |
| F3’E | 0.73 ± 0.23 | 0.42 ± 0.17 | 0.55 ± 0.12 | 0.65 ± 0.16 |
| WT | 1.00 ± 0.39 | 1.00 ± 0.39 | 1.00 ± 0.31 | 1.00 ± 0.28 |
| F3’G | 0.92 ± 0.34 | 0.41 ± 0.13 | 0.44 ± 0.12 | 0.80 ± 0.17 |
| F3’H | 0.88 ± 0.36 | 0.68 ± 0.34 | 0.80 ± 0.28 | 0.80 ± 0.28 |
| F3’I | 1.08 ± 0.35 | 0.65 ± 0.20 | 0.81 ± 0.21 | 0.82 ± 0.17 |
| F3’K | 0.80 ± 0.28 | 0.56 ± 0.20 | 0.84 ± 0.30 | 0.89 ± 0.30 |
| F3’L | 0.91 ± 0.29 | 0.59 ± 0.17 | 0.65 ± 0.18 | 0.81 ± 0.17 |
| F3’M | 0.73 ± 0.23 | 0.37 ± 0.14 | 0.66 ± 0.24 | 0.76 ± 0.31 |
| F3’N | 1.08 ± 0.35 | 0.49 ± 0.16 | 0.66 ± 0.15 | 0.79 ± 0.19 |
| F3’P | 0.78 ± 0.39 | 0.56 ± 0.30 | 0.42 ± 0.16 | 0.99 ± 0.45 |
| F3’Q | 1.16 ± 0.35 | 0.61 ± 0.18 | 0.79 ± 0.21 | 0.78 ± 0.22 |
| F3’R | 1.22 ± 0.11 | 1.11 ± 0.15 | 0.91 ± 0.05 | 1.14 ± 0.09 |
| F3’S | 1.22 ± 0.38 | 0.65 ± 0.20 | 0.82 ± 0.20 | 0.91 ± 0.20 |
| F3’T | 0.75 ± 0.25 | 0.47 ± 0.24 | 0.82 ± 0.28 | 0.65 ± 0.19 |
| F3’V | 1.21 ± 0.36 | 0.58 ± 0.20 | 0.81 ± 0.21 | 0.80 ± 0.21 |
| F3’W | 0.57 ± 0.20 | 0.41 ± 0.23 | 0.46 ± 0.12 | 0.70 ± 0.18 |
| F3’Y | 1.10 ± 0.34 | 0.84 ± 0.26 | 1.07 ± 0.26 | 0.97 ± 0.23 |
