# Supplementary material for: Profiling of Substrate Specificities of 3C-Like Proteases from Group 1, 2a, 2b, and 3 Coronaviruses
Source: PLoS One. 2011 Nov 2;6(11):e27228. doi: 10.1371/journal.pone.0027228 (PMC3206940; doi:10.1371/journal.pone.0027228)
Supplement: Table S2 — Correlation between activity of 3CLpro and structural properties of substituting residues. The correlation coefficients and p-values (bracketed) are reported. (DOC) [file pone.0027228.s003.doc]

**Table S2. Correlation between activity of 3CL^pro^ and structural properties of substituting residues. The correlation coefficients and p-values (bracketed) are reported.**

| **Position** | **Structural Property** | **HCoV-NL63** | **HCoV-OC43** | **SARS-CoV** | **IBV** |
| --- | --- | --- | --- | --- | --- |
| P5 | Volume | -0.183 (0.441) | -0.153 (0.519) | 0.221 (0.339) | 0.290 (0.214) |
|  | Hydrophobicity | 0.402 (0.079) | 0.559 (0.010) | 0.531 (0.016) | 0.521 (0.018) |
|  | α-helix | -0.256 (0.276) | -0.154 (0.517) | -0.103 (0.665) | -0.165 (0.487) |
|  | β-sheet | 0.347 (0.134) | 0.499 (0.025) | 0.689 (0.001) | 0.672 (0.001) |
| P4 | Volume | -0.527 (0.017) | -0.599 (0.005) | -0.412 (0.071) | -0.376 (0.102) |
|  | Hydrophobicity | 0.489 (0.029) | 0.425 (0.062) | 0.585 (0.007) | 0.517 (0.019) |
|  | α-helix | -0.076 (0.749) | -0.124 (0.603) | -0.139 (0.559) | -0.307 (0.188) |
|  | β-sheet | 0.199 (0.400) | 0.055 (0.819) | 0.307 (0.187) | 0.123 (0.605) |
| P3 | Volume | 0.085 (0.721) | 0.137 (0.565) | 0.351 (0.129) | 0.226 (0.339) |
|  | Hydrophobicity | 0.083 (0.729) | 0.055 (0.817) | 0.328 (0.159) | -0.004 (0.986) |
|  | α-helix | 0.044 (0.855) | 0.273 (0.245) | 0.241 (0.306) | 0.123 (0.605) |
|  | β-sheet | 0.391 (0.088) | 0.218 (0.356) | 0.581 (0.007) | 0.244 (0.300) |
| P2 | Volume | 0.177 (0.456) | 0.151 (0.524) | 0.245 (0.297) | 0.161 (0.496) |
|  | Hydrophobicity | 0.418 (0.067) | 0.547 (0.013) | 0.515 (0.020) | 0.507 (0.023) |
|  | α-helix | 0.258 (0.273) | 0.285 (0.223) | 0.366 (0.112) | 0.241 (0.306) |
|  | β-sheet | 0.209 (0.378) | 0.254 (0.279) | 0.250 (0.287) | 0.267 (0.255) |
| P1 | Volume | 0.031 (0.898) | 0.065 (0.785) | 0.034 (0.887) | 0.025 (0.918) |
|  | Hydrophobicity | -0.273 (0.244) | -0.248 (0.291) | -0.251 (0.285) | -0.270 (0.250) |
|  | α-helix | 0.108 (0.651) | 0.179 (0.450) | 0.131 (0.581) | 0.098 (0.681) |
|  | β-sheet | 0.021 (0.928) | 0.005 (0.983) | 0.029 (0.903) | 0.024 (0.919) |
| P1’ | Volume | -0.736 (<0.001) | -0.541 (0.014) | -0.687 (0.001) | -0.633 (0.003) |
|  | Hydrophobicity | 0.055 (0.818) | 0.379 (0.100) | 0.199 (0.401) | 0.280 (0.233) |
|  | α-helix | -0.386 (0.093) | -0.308 (0.186) | -0.250 (0.287) | -0.292 (0.212) |
|  | β-sheet | -0.260 (0.268) | 0.038 (0.873) | -0.194 (0.413) | -0.054 (0.820) |
| P2’ | Volume | -0.614 (0.004) | -0.532 (0.016) | -0.627 (0.003) | -0.351 (0.129) |
|  | Hydrophobicity | -0.083 (0.729) | 0.208 (0.379) | 0.037 (0.878) | -0.012 (0.958) |
|  | α-helix | -0.276 (0.240) | -0.171 (0.471) | -0.239 (0.311) | -0.137 (0.564) |
|  | β-sheet | -0.199 (0.401) | 0.049 (0.838) | -0.116 (0.626) | 0.127 (0.595) |
| P3’ | Volume | -0.069 (0.773) | 0.302 (0.195) | 0.432 (0.057) | 0.266 (0.256) |
|  | Hydrophobicity | 0.144 (0.545) | 0.079 (0.741) | 0.087 (0.716) | 0.000 (1.000) |
|  | α-helix | -0.228 (0.333) | -0.260 (0.269) | -0.070 (0.770) | -0.416 (0.068) |
|  | β-sheet | 0.371 (0.107) | 0.344 (0.138) | 0.538 (0.015) | 0.199 (0.400) |
