# Supplementary material for: Profiling of Substrate Specificities of 3C-Like Proteases from Group 1, 2a, 2b, and 3 Coronaviruses
Source: PLoS One. 2011 Nov 2;6(11):e27228. doi: 10.1371/journal.pone.0027228 (PMC3206940; doi:10.1371/journal.pone.0027228)
Supplement: Table S3 — Autocleavage sequences of 3CLpro. PEDV, TGEV, MHV, PHEV stand for porcine epidemic diarrhoea coronavirus, transmissible gastroenteritis coronavirus, mouse hepatitis coronavirus and porcine hemagglutinating encephalomyelitis coronavirus respectively. (DOC) [file pone.0027228.s004.doc]

**Table S3.** Autocleavage sequences of 3CL^pro^. PEDV, TGEV, MHV, PHEV stand for porcine epidemic diarrhoea coronavirus, transmissible gastroenteritis coronavirus, mouse hepatitis coronavirus and porcine hemagglutinating encephalomyelitis coronavirus respectively.

|  | **Group 1** | | | | **Group 2a** | | | | **Group 2b** | **Group 3** |
| --- | --- | --- | --- | --- | --- | --- | --- | --- | --- | --- |
| **Position** | **HCoV-NL63** | **HCoV-229E** | **PEDV** | **TGEV** | **HCoV-OC43** | **HCoV-HKU1** | **MHV-A59** | **PHEV** | **SARS-CoV** | **IBV** |
| NSP4↓5 | NSTLQ↓SGL | GSTLQ↓AGL | NSTLQ↓AGL | NSTLQ↓SGL | TSFLQ↓SGI | TSFLQ↓SGI | TSFLQ↓SGI | TSFLQ↓SGI | SAVLQ↓SGF | VSRLQ↓SGF |
| NSP5↓6 | GVNLQ↓SGK | GVNLQ↓SGK | GVNLQ↓GGY | GVNLQ↓AGK | GIKLQ↓SKR | GVKLQ↓SKT | GVKLQ↓SKR | GIKLQ↓SKR | GVTFQ↓GKF | GVRLQ↓SSF |
| NSP6↓7 | ANGLH↓APH | VSTVQ↓SKL | ISSVQ↓SKL | ISTVQ↓SKL | VSQFQ↓SKL | VSQIQ↓SKL | VSQIQ↓SRL | VSQFQ↓SKL | VATVQ↓SKM | IATVQ↓AKL |
| NSP7↓8 | SSTLQ↓SVA | DSILQ↓SVA | NSMLQ↓SVA | TTILQ↓SVA | NTVLQ↓ALQ | STVLQ↓ALQ | LQALQ↓SEF | LQALQ↓SEF | RATLQ↓AIA | STVLQ↓SVT |
| NSP8↓9 | VVKLQ↓NNE | VVKLQ↓NNE | IVKLQ↓NNE | TTKLQ↓NNE | ATVLQ↓NNE | NAVMQ↓NNE | TVVLQ↓NNE | ATVLQ↓NNE | AVKLQ↓NNE | DVVLQ↓NNE |
| NSP9↓10 | TIRLQ↓AGK | TVRLQ↓AGK | TVRLQ↓AGK | TVRLQ↓AGK | TVRLQ↓AGT | TIRLQ↓AGV | TVRLQ↓AGT | TVRLQ↓AGT | TVRLQ↓AGN | VVVLQ↓SKG |
| SP10↓12 | RTTIQ↓SVD | RTAIQ↓SFD | RSIMQ↓STD | RTSMQ↓SST | DTTVQ↓SKD | SVAVQ↓SKD | GSQFQ↓SKD | DTTVQ↓SKD | EPLMQ↓SAD | KSSVQ↓SVA |
| NSP12↓13 | STILQ↓AAG | STVLQ↓AAG | SAVLQ↓SAG | STVLQ↓AAG | SAVMQ↓SVG | SAVMQ↓SVG | SAVLQ↓SVG | SAVMQ↓SVG | HTVLQ↓AVG | PTTLQ↓SCG |
| NSP13↓14 | HADLH↓SSQ | MTDLQ↓SES | LSDLQ↓ANE | KIGLQ↓AKP | ETKVQ↓CST | LPRLH↓CTT | NPRLQ↓CTT | ETKVQ↓CST | VATLQ↓AEN | ETSLQ↓GTG |
| NSP14↓15 | ETNLQ↓SLE | EVNLQ↓GLE | SNNLQ↓GLE | SKALQ↓SLE | FTKLQ↓SLE | FTTLQ↓SLE | FTRLQ↓SLE | FTKLQ↓SLE | FTRLQ↓SLE | FSALQ↓SID |
| NSP15↓16 | YPQLQ↓SAE | YPQLQ↓SAE | YPQLQ↓ASE | YPQLQ↓SAE | YPRLQ↓AAS | YPKMQ↓ATN | YPRLQ↓AAA | YPRLQ↓AAS | YPKLQ↓ASQ | YPQLQ↓SAW |
